# 

**Published:** 1920-01-24

**Authors:** 


### A New Use for Anæsthetics.

THE Right Hon. A. J. Balfour, M.P., presided at a demonstration by Sir Jagadis Chandra Bose, given recently at the India Office. The lecturer gave an account of his investigations into the secrets of plant life, and of the discoveries he has made therein. By means of an instrument known as the Crescograph the highest powers of the microscope were magnified 100,000 times, and by these means one could actually witness the growth of plant life. Sir Jagadis had used anæsthetics on several occasions to assist him in the successful transplantation of trees. Such transplanted trees were wont to shed their leaves in the summer instead of the autumn, but this only continued for a time; in due course the trees would behave in a normal manner. The lecturer considered the fundamental unity of life reactions, which he was able to demonstrate between animals and plants, to be of the very greatest importance. Sir Jagadis had observed spontaneous pulsation in certain plant tissues which were comparable to the animal heart-beat. The actions of many stimulants, anæsthetics, and poisons also seemed identical in both animal and vegetable tissues.

### A Municipal Laboratory.

BIRMINGHAM is to establish a municipal laboratory. The Corporation is terminating an old arrangement with the authorities of Birmingham University for the necessary bacteriological work of the public health department, and is now arranging to do the work municipally. The Corporation, having failed to rent suitable premises, has now arranged to fit one of the wards at Lodge Road Hospital for a laboratory. Mr. Herbert Henry, M.D., has been appointed bacteriologist at a salary of £700 a year, and will begin his work early in the New Year.

### Unfit Houses and Unhealthy Areas.

SUCH is the title of a new issue by the Ministry of Health, and is a manual to assist local authorities and others concerned in the "Housing Problem." One of the most difficult problems of the present day is the efficient clearing of slum areas preparatory to the erection of new buildings in their place. In this regard the Ministry advises that each local authority makes a careful survey of the area within its jurisdiction, and then submits proposals for dealing with the unfit houses and unhealthy areas disclosed in the survey. The speed with which such proposals will be dealt with depends upon the acuteness of house shortage in the district surveyed.

### Gifts and Bequests.

HUDDERSFIELD Royal Infirmary has been left £100 under the will of the late Mr. Edward Schofield.

ALDERMAN A. C. ROGERS, of Buckingham, has left £300 to the Buckingham Nursing Home.

ST. BARTHOLOMEW'S HOSPITAL has been left £1,000 and the Great Northern Central Hospital £500 under the will of Mr. Arthur Hill, of the Stock Exchange.

UNDER the will of the late Mr. J. H. Beddington, of Sussex Place, S.W., £100 each has been left to the London Hospital and University College Hospital.

THE PRINCE OF WALES has sent the Great Northern Central Hospital a cheque for £405, being one-third of the proceeds of the display at the Royal Albert Hall of the film of His Royal Highness's Canadian tour.

MAJOR-GENERAL SIR ALBERT WILLIAMS, K.C.V.O., has left £100 each to the Cancer Hospital, St. George's Hospital, the Dorset Hospital, and the Soldiers' Daughters' Home, Hampstead.

DR. SCOWCROFT, who has been associated with the Manchester Royal Infirmary for forty years, has presented about 52 acres of land in front of the Cheadle Asylum to the Board of Management in order that an open space shall be kept permanently in front of the asylum.

THE Chelsea Hospital for Women has received £500 from the executors of the late Mr. Aubrey Robinson out of the charitable legacy left by his will, also £100, second instalment, of the late Mrs. Stephenson's bequest, and £100 bequest from the late Mrs. M. W. Clark.

To commemorate the advent of peace, and as a thanksgiving for victory, the National Deposit Friendly Society has given £1,000 to the Hospital for Sick Children, Great Ormond Street, for the endowment of a cot, to bear the name of the Society.

MRS. HANNAH PLAYER, of Bristol, has bequeathed £500 to the Bristol General Hospital, £250 each to the Eye Hospital and the Royal Hospital for Sick Children and Women. The residue of the estate has been left to Bristol Royal Infirmary.

MR. WILLIAM CARNELLY, late chairman of Messrs. Rylands and Sons, Ltd., of Manchester, died recently at the age of ninety-six, and left a number of legacies to charities, amongst which are £2,000 to the Beckett Hospital, £1,000 to the Manchester Royal Infirmary, £400 to St. Bartholomew's Hospital, London, and £300 to Guy's Hospital.

MR. G. T. WADE, an auctioneer, of Leicester, has left £1,500 each to the Leicester Infirmary and the Children's Hospital to endow a bed and cots; £1,000 each to St. Luke's Home for the Dying, the Cancer Hospital, Charnwood Convalescent Home, and the Royal Society for the Prevention of Cruelty to Children; and £500 each to the Blind Institution, Leicester, and the Royal Hospitals for Incurables, Leamington and Putney.

### Personalia.

### Lady Assistant Medical Officer.

MISS ARABELLA CRORY KIRKER, junior medical officer at the Royal Waterloo Hospital for Women and Children, has been appointed lady assistant medical officer of health by the Southwark Borough Council. Miss Kirker's duties will be principally confined to child-welfare work. The appointment is a whole-time one, with a commencing salary of £400 a year, rising by annual increments of £50 to a maximum of £500. Miss Kirker holds the diplomas of M.B. Bet, B.A.O., Queen's University, Belfast, and was house surgeon at the Royal Victoria Hospital, Belfast, with charge of surgical and ophthalmic beds. After being in civil medical practice in Birmingham, she came to the Royal Waterloo Hospital in July.

### New Buildings Contemplated.

**The New Westminster Hospital.**—*The Builder* New Year's Number reproduces a large drawing of the proposed new Westminster Hospital, of 525 beds, Clapham Common, from the design of the architects, Messrs. H. P. Adams and C. Holden. The existing building frontage lines on the south side have been retained, thus allowing of a strip of garden between the ends of the wards on this side and the public road. The blocks are all reached on both sides from one main central corridor, and the administration offices are arranged midway between the floor pavilions. The out-patient department, on the ground floor of the north-east pavilion, consists of a waiting-hall for 250 patients, six consulting-rooms, operating-theatre, and other rooms. The casualty department is in the south-east pavilion, with the electrical department, gymnasium, etc., below in the basement. The boiler-house and engine-rooms are under the out-patient department. The ward pavilions are 80 feet apart, and placed almost axially north and south; they are three storeys high, each floor containing twenty-four beds, having a south balcony and the usual off-rooms. There are four operating-theatres, with due north and top lighting. Provision has been made for a large medical school, and in the future for a nurses' home.

BALLYRATTEN.—Mr. J. M. Robinson has been appointed architect for the proposed intercepting hospital at Ballyratten, Moville, to be built for the Londonderry Port Authority.

INSCH.—A site given by Mr. C. E. N. Leith Hay has been accepted for the Memorial Hospital, and plans will be drawn up as early as possible.

JARROW.—The Town Council has decided to obtain an estimate of the cost for providing a maternity hospital.

PONTSARN.—Mr. H. Seymour Berry, of Merthyr, has offered to give a site at Pontsarn, near Merthyr Tydvil, and spend £10,000 on temporary hospital buildings. He is also prepared to contribute £10,000 towards a new permanent general hospital, provided the public establish an endowment fund of £100,000.

TOWYN.—Tenders are invited for the erection of a Memorial Cottage Hospital. Specifications may be obtained from Mr. F. Howarth, L.R.I.B.A., Architect, Towyn.

COLWYN BAY.—The British Red Cross Society has promised a grant of £10,000 towards the £25,000 needed for the proposed general hospital.

CONGLETON.—Mr. J. H. Walter, the borough surveyor, has been appointed architect for the new War Memorial Hospital.

EDENBRIDGE.—The local War Memorial Committee has instructed Mr. Little, of Tonbridge, to prepare plans and specifications for the proposed cottage hospital.

HALTWHISTLE.—A sum of £6,154 has been raised for a new hospital.

HAYLE.—It is proposed to provide an isolation hospital in close proximity to the port of Hayle, and local authorities are considering co-operation.

HIGH WYCOMBE.—Mr. Wallace Marchment and Mr. Horace Cubitt are joint authors of the first premiated design in the hospital competition

LLANDRINDOD WELLS.—Major Gibson-Watt has presented the County Hospital Committee with a valuable site of three acres for a new county hospital, which it is proposed to build as a war memorial.

### MEDICAL APPOINTMENTS.

KING'S COLLEGE HOSPITAL AND MEDICAL SCHOOL.

The following appointments have been made to the Staff: H. W. WILTSHIRE, D.S.O., O.B.E., M.A., M.D., F.R.C.P., Physician-in-Charge of the Cardiological Department, Lecturer on Cardiology, and Vice-Dean of the Medical School. F. W. TUNNICLIFFE, M.D., M.R.C.P., Physician-in-Charge of the Therapeutics and Applied Pharmacology Department, and Lecturer on Therapeutics and Applied Pharmacology. A. C. D. Firth, M.A., M.D., M.R.C.P., Junior Physician and Demonstrator of Clinical Medicine. R. H. STEEN, B.A., M.D., M.R.C.P., Out-patient Physician for Psychological Medicine. S. A. KINNIER WILSON, M.A., M.D., F.R.C.P., Junior Neurologist and Lecturer on Neurology. J. W. THOMSON WALKER, O.B.E., M.B., C.M., F.R.C.S., Senior Urologist and Lecturer on Urology. H. A. T. FAIRBANK, D.S.O., M.S., F.R.C.S., Senior Orthopædist and Lecturer on Orthopædics. JOHN EVERIDGE, O.B.E., F.R.C.S., Junior Urologist and Junior Surgeon, Lecturer on Surgical Applied Anatomy. C. JENNINGS MARSHALL, M.D., M.S., F.R.C.S., Junior Orthopædist and Junior Surgeon, Lecturer on Surgical Applied Anatomy. W. J. R. SIMPSON, C.M.G., M.D., F.R.C.P., D.P.H., Lecturer on Tropical Medicine. W. D'ESTE EMERY, M.D., B.Sc., M.R.C.P., Pathologist, Director of Pathological Department and Lecturer on General Pathology. E. B. CLAYTON, M.B., B.C., M.R.C.S., Physico-therapist and Director of Physico-therapeutic Department. L. M. MOODY, M.D., B.S., M.R.C.P., Bacteriologist and Lecturer on Bacteriology. G. A. HARRISON, B.A., M.R.C.S., Bio-chemist and Lecturer on Medical Chemistry. HELEN INLEBY, M.B., B.S., M.R.C.P., Pathological Registrar and Lecturer on Morbid Anatomy. H. A. BURRIDGE, M.A., M.B., M.R.C.S., Lecturer on Forensic Medicine and Toxicology. J. H. SHELDON, M.B., B.S., M.R.C.S., Sambrooke Medical Registrar and Medical Tutor. C. P. G. WAKELEY, M.R.C.S., Sambrooke Surgical Registrar and Surgical Tutor. A. C. McALISTER, M.B., B.S., M.R.C.S., Obstetric Registrar and Obstetric Tutor.

The Royal Sussex County Hospital, Brighton.—Mr. T. H. Ionides, F.R.C.S., has been appointed honorary surgeon, *vice* Mr. A. H. Buck, F.R.C.S.; Mr. A. Geoffery Bate, F.R.C.S., honorary assistant surgeon, *vice* Mr. T. H. Ionides.

Dr. E. Colston Williams, M.D. (Lond.), F.R.C.S.Edin., Brecon, has been appointed medical officer of health for the County of Glamorgan, at a salary of £1,000 a year. He was a student at St. Bartholomew's Hospital, where he gained three scholarships and other prizes. After graduating M.D. at London University, and eventually taking his F.R.C.S., Dr. Williams held appointments at St. Luke's Mental Hospital, Great Ormond Street, East London Children's Hospital, and the Hospital for Nervous Diseases, Queen Square.

### MATRONS' AND SISTERS' APPOINTMENTS.

Bishops Stortford Infectious Hospital, Haymeads.—K. B. Langston has been appointed nurse-matron. She was trained at Birmingham City Hospital and at South Manchester Hospital, and has since been nurse-matron of Faversham Isolation Hospital and of Chippenham Joint Isolation Hospital. She is a member of the College of Nursing.

Borough Hospital, Birkenhead.—Miss A. Wilson and Miss I. Hill have been appointed sisters. Miss Wilson was trained at St. Marylebone Infirmary, has been nurse at the Metropolitan Hospital for Children, Broadstairs, the Infirmary, Maidenhead, head nurse at St. Peter's Home, Woking, and sister at the Red Cross Hospital, Maidenhead. She has also done private nursing, and holds the certificate of the Central Midwives Board. Miss Hill was trained at the City and District Infirmary, York, and at the General Infirmary, Huddersfield. She has been sister at the first-named institution and staff nurse and sister in Queen Alexandra's Imperial Military Nursing Service Reserve.

Cottage Hospital, Bexley Heath.—Miss Beatrice Alcock has been appointed matron. She was trained at Coventry and Warwickshire Hospital, and has since been night superintendent at the Royal Hospital, Richmond, Surrey; sister at the Kendray Fever Hospital, Barnsley, at the Royal Infirmary, Penzance; sister and deputy-matron at Stroud General Hospital; sister-in-charge of Park Lodge Nursing Home, Brockley. She served in the Territorial Force Nursing Service at the 5th Southern General Hospital, and was acting matron of the Manoel Hospital, Malta. She has been awarded the Royal Red Cross, Second Class.

County Borough Maternity Hospital, Swansea.—Miss Elizabeth Crompton has been appointed matron. She was trained at Manchester Royal Infirmary, and has since been sister-in-charge of the maternity block and labour ward of the Willesden Municipal Hospital.

Manchester Ear Hospital.—Miss Mysie Hobson has been appointed theatre sister. She was trained at the Royal Victoria Hospital, Belfast, and has been theatre sister at the Lord Derby War Hospital, Warrington.

Peasley Cross Sanatorium, St. Helens, Lancashire.—Miss Rebecca Linehan has been appointed sister. She was trained at the above-named institution and at the Royal Devon and Exeter Hospital, where she has since been on the private staff.

Poor-Law Infirmary, Barton-on-Irwell.—Miss N. Dolan has been appointed superintendent nurse. She was trained at the Walton Infirmary, Liverpool, where she also gained the certificate of the Central Midwives Board, and where she was later sister, theatre sister, and night superintendent.

Queen's Hospital, Birmingham.—Miss Eva G. Thomas has been appointed sister. She was trained at Bristol Royal Infirmary, has been sister there and at the Cossham Memorial Hospital, Kingswood, Bristol, and ward and night sister at the 2nd Southern General Hospital. Miss Thomas holds the certificate of the Incorporated Society of Trained Masseuses.

Ruthin Hospital.—Miss Irene L. Jones has been appointed matron. She was trained at Bootle Borough Hospital, where she was later night sister and matron's assistant. She has also been ward sister at Hartlepool Hospital, at the Royal Eye and Ear Hospital, Bradford, night superintendent at the Royal National Orthopædic Hospital, and matron of the Leaf Hospital, Eastbourne, the Liverpool Skin Hospital, and the Cottage Hospital, Mold.

Woolwich Union Infirmary, Plumstead.—Miss Lucy Smith has been appointed ward sister. She was trained at West Bromwich Infirmary, where she was later sister of the female surgical ward and night superintendent. She has since been sister to Birmingham County Council, health visitor for West Riding County Council, head sister at Holbeck Union Infirmary, and has served as sister for military work.

### The College of Nursing
### (Limited by Guarantee).

The College of Nursing has appointed a Committee to consider the Hours of Employment Bill.

